# Involvement of inflammatory responses in the early development of calcific aortic valve disease: lessons from statin therapy

**DOI:** 10.1080/19768354.2018.1528175

**Published:** 2018-09-28

**Authors:** Seung Hyun Lee, Jae-Hoon Choi

**Affiliations:** Department of Life Science, College of Natural Sciences, Research Institute for Natural Sciences, Hanyang University, Seoul, Republic of Korea

**Keywords:** Calcific aortic valve disease, inflammation, immune cells, statin

## Abstract

Calcific aortic valve disease (CAVD) is the most common degenerative heart valve disease. Among the many risk factors for this disease are age, hypercholesterolemia, hypertension, smoking, type-2 diabetes, rheumatic fever, and chronic kidney disease. Since many of these overlap with risk factors for atherosclerosis, the molecular and cellular mechanisms of CAVD development have been presumed to be similar to those for atherogenesis. Thus, attempts have been made to evaluate the therapeutic efficacy of statins, representative anti-atherosclerosis drugs with lipid-lowering and anti-inflammatory effects, against CAVD. Unfortunately, statins have shown little or no effect on CAVD development. But some reports suggest that statins may prevent or reduce the development of early stage CAVD in which having calcification is absent or minimal. These results suggest that therapeutic approaches should differ according to the stage of disease, and that a precise understanding of the mechanism of aortic valve calcification is required to identify novel therapeutic targets for advanced CAVD. Given the involvement of inflammatory processes in the development and progression of CAVD, current therapeutic approaches for chronic inflammatory cardiovascular disease like atherosclerosis may help to prevent or minimize the early development of CAVD. In this review, we focus on several inflammatory cellular and molecular components involved in CAVD that might be considered drug targets for preventing CAVD.

## Introduction

Calcific aortic valve disease (CAVD) is a degenerative disease characterized by calcification, inflammation and fibrosis, leading to hardening of the aortic valve leaflet, orifice narrowing and stenosis. This pathological process can lead to left ventricular overload, which can cause death. It is estimated that about 2.5 million people worldwide currently suffer from CAVD. Non-invasive commercial medications to treat CAVD do not yet exist, and the only definitive treatment is invasive aortic valve replacement (AVR). If AVR is not properly performed after manifestation of symptoms, CAVD is a highly lethal disease with 2- and 5-year survival rates of 50% and 25%, respectively (Otto 2000; Dweck et al. 2012; Nishimura et al. 2014; Otto and Prendergast 2014). Several previous reports based on in vitro and in vivo experiments and human clinical data have suggested that hyperlipidemia and inflammatory responses caused by oxidative modification of lipoproteins play pivotal roles in CAVD as well as atherosclerosis (Olsson et al. 1999; Rajamannan 2006). These observations suggest that the disease-onset mechanism of CAVD may be similar to the early mechanism of atherosclerosis.

Statins – HMG-CoA reductase inhibitors – , which have been used clinically to decrease plasma lipid levels and attenuate atherosclerosis, also have pleiotropic activities, including anti-oxidative effects, modulation of endothelial function, and regulation of inflammatory processes. These observations have prompted researchers to evaluate the efficacy of statins on the progression of CAVD. Although statins have shown some effectiveness at the early stage of valvular sclerosis, no beneficial effects have been observed against mid-late stage CAVD (Hutcheson et al. 2014). These results strongly suggest that, although the early pathogenesis of disease progression is similar for CAVD and atherosclerosis, mid-late stage diseases develop through different pathways. Because of these CAVD features, numerous studies have been conducted to understand the development and progression of CAVD and identify new therapeutic targets.

In this review, we first focus on the cellular components of CAVD and describe how valvular interstitial cells (VICs), endothelial cells, and various immune cells are involved in disease progression. Second, we discuss the role of a number of humoral factors, including cytokines, angiotensin II and lipoprotein(a) [Lp(a)], in the onset and progression of CAVD, including their involvement in inflammation and calcification.

## Valvular interstitial cells

VICs, a major cell type in valves, contribute to aortic valve sclerosis and stenosis through various processes. Under normal conditions, VICs play an important role in valvular homeostasis. However, during disease, VICs can differentiate into myofibroblast or osteoblast-like cells in manner that depends on tumor growth factor (TGF)-β, leading to aortic valvular fibrosis, calcification, and stenosis (Weiss et al. 2013; Wang et al. 2014; Lee and Choi 2016). VIC-mediated calcification, similar to bone formation, is mainly induced by bone morphogenic proteins (BMPs). In this process, transcription factors, including runt related transcription factor 2 (Runx2), nuclear factor of activated T cells 1 (NFATc1) and osterix, are activated, resulting in an increase in the expression of osteopontin and BMPs. Importantly, BMP2 promotes calcification by increasing alkaline phosphatase (ALP) expression via the Wnt/β-catenin pathway (Rajamannan et al. 2003; Alexopoulos et al. 2010; Leopold 2012). In addition, VICs can induce inflammatory responses in diseased aortic valves. VICs express receptors for several pathogen-associated molecular patterns (PAMPs), including the Toll-like receptors, TLR2, TLR3 and TLR4. Activation of these receptors by agonist not only induces Runx2 and BMP2 expression, it also activates *nuclear factor kappa B (NF-κB)*, which is an important transcription factor in inflammation. Activation of NF-κB can increase the production of inflammatory cytokines, leading to exacerbation of aortic stenosis (Tak and Firestein 2001; Meng et al. 2008; Mathieu et al. 2015; Zhan et al. 2015). In particular, interleukin (IL)-6, a major VIC cytokine, is increased in patients with various cardiovascular diseases and plays a role in BMP2-induced valvular calcification (Kanda and Takahashi 2004; Mathieu et al. 2015). Moreover, VICs that have differentiated into myofibroblasts in CAVD express receptors, including CD36 and LOX-1 (lipoxygenase 1), capable of binding oxidized low-density lipoprotein (oxLDL). These cells respond to oxLDL by producing monocyte chemoattractant protein (MCP)-1, IL-6, IL-8 and macrophage colony-stimulating factor (M-CSF), but reduce their production of osteoprotegerin, a factor that inhibits vascular calcification. Thus, oxLDL can induce valvular inflammation and calcification through interactions with VICs (Syvaranta et al. 2014).

Valvular endothelial cells (VECs), another important cellular component of valves, also play various roles in the progression of CAVD. Under normal conditions, VECs contribute to the maintenance of valvular homeostasis together with VICs. But in disease states, VECs can be affected by changes in shear stress such that altered mechanical forces induce the expression of BMP4, TGF-β and their effector genes, including vascular cell adhesion protein (VCAM)-1 and intercellular adhesion molecule (ICAM)-1 (Sucosky et al. 2009).

Indeed, unlike the case under normal conditions, aortic VECs in the valves of CAVD patients express high levels of cell adhesion molecules such as VCAM-1, ICAM-1, and E-selectin. Expression of these adhesion molecules induces the recruitment of immune cells, including monocytes and T cells, into diseased valves and enhances inflammatory processes (Ghaisas et al. 2000; Muller 2013). Interestingly, gene expression profiles induced by shear stress are markedly different between endothelial cells in valves and those in the aorta. Several genes involved in calcification and inflammation are differentially expressed in these endothelial cells and are further differentially affected by shear stress. These results suggest that phenotypic changes of endothelial cells in valves caused by altered shear stress may be different from the changes observed in aortic endothelial cells. Moreover, the gene expression profiles of VECs in aortic and ventricular sites may also differ because of differences in shear stress (Butcher et al. 2004; Butcher et al. 2006; Butcher and Nerem 2007; Mahler et al. 2014).

## Immune cells

To date, a number of different immune cells have been demonstrated to play roles in the pathogenesis of aortic valve sclerosis and stenosis. During the progression of CAVD, circulating leukocytes, including T cells and monocytes, are recruited to valves, where infiltrated monocytes differentiate into macrophages (Otto et al. 1994; Wallby et al. 2002; Aikawa et al. 2007; Steiner et al. 2012). The recruitment of immune cells is thought to be caused by upregulation of cell adhesion molecules such as ICAM-1 and VCAM-1 by oxLDL, and by endothelial damage (Ghaisas et al. 2000; Miller et al. 2011). In addition to T cells and monocytes/macrophages, dendritic cells (DCs), B cells, and mast cells are involved in the progression of CAVD through interactions between VICs and VECs, leading to inflammation and calcification, and thereby worsening CAVD (Weiss et al. 2013; Mathieu et al. 2015; Lee and Choi 2016).

Macrophages, a major phagocytic cell type, take up lipids and differentiate into foam cells in atherosclerosis. Similarly, macrophages can take up lipids and form foam cells in aortic valve sclerosis and stenosis. VICs also take up lipids, but their uptake ability is less than that of macrophages. In CAVD, foamy macrophages are localized near the annulus area of the valve (Filip et al. 1987; Mehrabian et al. 1991). Macrophages can secrete various proinflammatory molecules, including tumor necrosis factor (TNF)-α, IL-1β, matrix metalloproteinases (MMPs), and cathepsins. These proinflammatory molecules activate VICs and enhance inflammation and calcification. MMPs and cathepsins directly induce remodeling of the extracellular matrix, which is highly involved in exacerbating calcification (Kaden et al. 2003; Kaden, Dempfle et al. 2005; Kaden, Kilic et al. 2005; Aikawa et al. 2007; Aikawa et al. 2009; Arango Duque and Descoteaux 2014).

T cells, like macrophages, are presumed to produce several inflammatory cytokines that lead to activation of VICs in CAVD (Huse et al. 2006; Dweck et al. 2012). According to previous reports, oligoclonal CD4+ and CD8+ T cells accumulate in stenotic aortic valves, and CD8 + CD28- effector memory T cells are elevated in blood and valve tissue of these patients. Accordingly, T cells in stenotic aortic valves are generated by antigen-specific clonal expansion, and T-cell clones specific to common antigens may be found in CAVD patients, indicating that systemic adaptive immunity is integrally involved in the progression of CAVD.

Defining antigens specific to the T-cell receptor (TCR) of these clonal T cells is important to develop new therapeutic approaches (Wu et al. 2007; Winchester et al. 2011). Understanding the roles of regulatory T cells (Tregs) in CAVD is also thought to be necessary in this context. Circulating Tregs are increased in the blood of patients with CAVD, but their function in the valve itself remains to be elucidated. Future studies should determine whether Tregs reduce CAVD, as they do in other cardiovascular diseases, through immune regulatory functions, or enhance CAVD by producing TGF-β, leading to calcification and fibrosis (Branton and Kopp 1999; Clark-Greuel et al. 2007; Meng et al. 2016; Shimoni et al. 2016). Recently, Nagy and colleagues provided new functional insights into the relationship of CD8+ T cells to calcification in stenotic aortic valves, suggesting that infiltrated CD8+ T cells produce interferon (IFN)-γ, leading to a reduction in the number and function of osteoclasts, which may enhance valve calcification (Nagy et al. 2017). These results indicate that T cells play important roles in the pathogenesis of CAVD through various mechanisms in addition to their role in immune responses. B cells, another type of adaptive immune cell, and its active form, the plasma cell, have also been reported to accumulate in stenotic aortic valves, but the exact function of B cells in CAVD remains to be elucidated (Steiner et al. 2012; Natorska et al. 2016).

Mast cells are known to play important roles in aortic valve stenosis as well as various cardiovascular diseases, including myocardial infarction, atherosclerosis, and aortic aneurysm (Bot et al. 2008; Wypasek et al. 2013). Importantly, mast cells produce chymase, which converts angiotensin I to angiotensin II. Local production of angiotensin II in stenotic aortic valves by mast cell-derived chymase is involved in aortic valve stenosis and thus contributes to disease progression (Urata et al. 1990; Helske et al. 2004). In addition, mast cells induce tryptase, which can downregulate endostatin and produce vascular endothelial growth factor (VEGF)-A, leading to increased angiogenesis in the stenotic valve (Syvaranta et al. 2010). Activated mast cell also exacerbate aortic stenosis by producing cathepsin G, which causes elastin degradation and tissue remodeling in aortic valves (Helske et al. 2006).

Interestingly, DCs, a potent antigen-presenting cell type, are abundant in aortic valves, and a majority of DCs reside under the endothelium on the aortic side of the aortic valve – a disease-prone area (Choi et al. 2009). Previously, Flt3 (fms-related tyrosine kinase 3)-dependent DCs have been shown to attenuate atherosclerosis (Choi et al. 2011). Thus, because DCs may control inflammatory processes in valves, future studies should explore the exact roles of DCs in CAVD.

## Angiotensin II

Angiotensin II, a pivotal component of the renin-angiotensin system, is a major contributor to aortic valve disease as well as other cardiovascular diseases (Helske et al. 2004; Ferrario 2006). Angiotensin II is produced by cleavage of angiotensin I by angiotensin-converting enzyme (ACE), and its effects are mediated by the angiotensin II receptors, AT1R and AT2R. AT1Rs are distributed throughout the cardiovascular system, including in vessels and the heart, and their activation by angiotensin II can aggravate cardiovascular diseases. (Ferrario 2006) ACE is detectable in plasma LDL from aortic valve sclerosis and stenosis patients, and is also found in valvular lesions. ACE in valve lesions partially co-localizes with macrophages, but the majority of ACE, as well as angiotensin II, is co-localized with ApoB in the extracellular region (O'Brien et al. 2002). Although AT1Rs are not expressed in normal aortic valves, they are expressed in myofibroblasts in the stenotic aortic valve (O'Brien et al. 2002). Activated mast cells, which are abundant in stenotic valves, also express considerable amounts of ACE and AT1R and secrete chymase, which can act as an angiotensin II-producing enzyme. Taken together, these observations indicate that systemic angiotensin II, as well as locally produced angiotensin II, underlie the progression of aortic stenosis (Urata et al. 1990; Helske et al. 2004; Wypasek et al. 2013). Engagement of the AT1R by angiotensin II directly activates immune cells through activation of NF-κB and leads to upregulation of several inflammatory mediators, including MCP-1, RANTES, IL-6, ICAM1, and VCAM1 (Suzuki et al. 2003). These findings have prompted tests of numerous inhibitors and blockers of angiotensin II pathway components, such as ACE and AT1R, in animals and other preclinical models. A previous report has shown that a high dose of angiotensin II induces aortic valve leaflet thickening, discontinuities in the endothelial lining, and an increase in myofibroblasts in the ApoE^-/-^ mouse model of atherosclerosis. These disease phenotypes are reduced by olmesartan, an AT1R blocker, but not by the anti-hypertensive agent hydralazine, indicating that angiotensin II actions on valvular sclerosis and stenosis are unrelated to blood pressure (Fujisaka et al. 2013). In addition, the ACE inhibitor enalapril attenuates valvular thickening in the partially nephrectomized ApoE^-/-^ mouse (Simolin et al. 2009). Olmesartan has also been shown to reduce accumulation of macrophages, expression of osteopontin, upregulation of ACE, and increases in myofibroblasts in aortic valves of rabbits fed a cholesterol diet (Arishiro et al. 2007). On the basis of this significant role of angiotensin II, blockers of the angiotensin II pathway have been developed as therapeutics for CAVD. Although initial therapeutic trials raised concerns about potential untoward side effects of angiotensin II pathway inhibitors, treatment with ACE inhibitors or AT1R blockers has shown improved survival rates compared with controls. If the effectiveness and safety of these blockers are confirmed in subsequent studies, it can be expected that these agents will be used clinically for the treatment of CAVD (Otto 2001; Routledge and Townend 2001; Shavelle 2005; Nadir et al. 2011).

## Cytokines

The proinflammatory cytokine, IL-1β, is increased in the stenotic aortic valve leaflet and enhances the expression of MMPs, leading to exacerbation of aortic valvular stenosis. IL-1β also activates the *NF-κB* pathway, leading to the production of IL-6, IL-8, and MCP-1 in human AVICs (Kaden et al. 2003; Nadlonek et al. 2013). IL-1Ra (IL-1 receptor antagonist), a member of the IL-1 family that, like IL-1β, also acts at the IL-1 receptor, can block binding of IL-1β to IL-1Rs, thereby attenuating the pro-inflammatory action of *IL-1β*. Consistent with this, an IL-1Ra deficiency has been shown to induce aortic valve thickening accompanied by increased macrophage infiltration into valves and elevated plasma levels of IL-1β, TNF-α, and IL-6. Similarly, mice transplanted with IL-1Ra^-/-^ bone marrow exhibit aortic valve thickening, as do nu/nu mice transplanted with T cells lacking IL-1Ra. These results indicate that IL-1Ra in bone marrow-derived immune cells, including T cells, has an important role in protecting against valvular thickening. It has also been shown that TNF-α is an important participant in the development of aortic stenosis in IL-1Ra^-/-^ mice (Arend et al. 1998; Isoda et al. 2010).

TGF-β increases ALP activity in VICs and also promotes apoptosis, leading to calcification. It also increases the expression of metalloproteinases, including MMP2 and MMP9 (Clark-Greuel et al. 2007). In addition, TGF-β enhances β-catenin–mediated differentiation of VICs into myofibroblasts in the fibrosa layer, which is a more lesion-prone area than the ventricularis layer. This process occurs in matrices with a stiffness comparable to that of the fibrosa layer of the valve, which is a lesion-prone area ^29.^ Although TGF-β is presumed to induce the differentiation of aortic valve endothelial cells into VICs or α-SMA (smooth muscle actin)-expressing myofibroblasts, it does not induce endothelial mesenchymal transition (EndMT) in adults, despite inducing the expression of α-SMA and Snail (Paranya et al. 2001; Mahler et al. 2013). Mice lacking Emilin1, which has been reported to reduce TGF-β signaling, not only exhibit activation of VICs, inflammation and fibrosis, they also show enhanced angiogenesis, indicating that TGF-β is involved in angiogenesis in aortic valvular disease (Munjal et al. 2014). By activating NF-κB in endothelial cells in the aortic valve, TNF-α and IL-6 induce EndMT, weakening of the endothelial lining, and differentiation of endothelial cells into VICs (Mahler et al. 2013). TNF-α, which is increased in the serum of aortic stenosis patients, is primarily expressed by infiltrating macrophages in the diseased valve, where it leads to induction of calcification through Cbfa-1 (Runx2) and consequent ALP activation (Kapadia et al. 2000; Kaden, Dempfle et al. 2005; Kaden, Kilic et al. 2005). This process is dependent on Runx2 and Dlx5 (distal-less homeobox 5), reflecting the fact that TNF-α activates NF-κB and BMP2 pathways (Yu et al. 2011; Galeone et al. 2013). IL-6 is known to be *involved* in various cardiovascular diseases, and its serum levels are increased in CAVD. In the aortic valve itself, IL-6 is expressed to a greater extent in the calcified valve than the non-calcified valve and appears to increase calcification. The effects of IL-6 on valvular calcification are negatively regulated by P2Y2 purinergic receptor signaling, which functions to repress Akt/NF-κB activation. In the diseased valve, the expression of ENPP1, which suppresses P2Y2 receptor-mediated signaling by breaking down ATP into AMP and inorganic phosphate (Pi), is elevated, accounting for the increase in IL-6 production (Kanda and Takahashi 2004; Cote et al. 2012; El Husseini et al. 2014; Mathieu et al. 2015).

Zeng and colleagues recently reported that IL-37 expression is reduced in VICs in the diseased human aortic valve compared with that in the normal aortic valve. IL-37 attenuates the expression of BMP2 and ALP induced by TLR2 and TLR4 stimulation in human aortic VICs, leading to attenuation of osteogenic responses, including calcification and subsequent aortic valvular thickening. Moreover, IL-37 effectively decreases osteogenic responses induced by exposure to oxLDL. This anti-osteogenic role of IL-37 in human aortic VICs is dependent on the phosphorylation (activation) of NF-κB and extracellular signal-regulated kinase (ERK)-1/2 (Zeng et al. 2017).

In 2009, Bosse and colleagues analyzed gene expression profiles from five normal aortic valves and five stenotic valves using Affymetrix GeneChips. These analyses showed that stenotic aortic valve express higher levels of various cytokines, chemokines and related genes, including IL-1β and CCL4, and exhibit highly activated TGF and TNF signaling. These results suggest that aortic valve stenosis is a dynamic disease induced by the actions of cytokines and chemokines (Bosse et al. 2009).

## Lipoprotein(a)

Lp(a), encoded by *LPA*, has been characterized as a risk factor for cardiovascular diseases.(Nordestgaard et al. 2010) In particular, Lp(a) has been presumed to be an important genetic risk factor for aortic valve stenosis, for which the only available treatment option is AVR (Thanassoulis 2016; Yeang et al. 2016). Various epidemiology studies have shown that Lp(a) levels are increased in patients with aortic valve stenosis and are highly positively correlated with aortic valve stenosis (Gotoh et al. 1995; Stewart et al. 1997; Kamstrup et al. 2014).

Extensive genetic studies have sought to establish a relationship between single-nucleotide polymorphisms (SNPs) in the *LPA* gene and aortic valve stenosis. In 2013, Thanassoulis and colleagues performed genome-wide screening on 6942 patients diagnosed with aortic valve calcification based on computed tomography (CT) scans, and identified an SNP (rs10455872) in the *LPA* gene in chromosome 6 that was significantly correlated with aortic valve stenosis (Thanassoulis et al. 2013). Arsenault and colleagues subsequently demonstrated that the G allele of *LPA* rs10455872 was correlated with elevated serum Lp(a); that is, individuals homozygous for the GG allele showed higher serum Lp(a) levels than AG heterozygotes, which, in turn, showed higher serum Lp(a) levels than individuals homozygous for the common allele, an observation that accounts for the correlation between this SNP and aortic valve stenosis (Arsenault et al. 2014). In addition to these associations with SNP rs10455872, a smaller number of Kringle IV type-2 (KIV-2) repeats in the *LPA* gene was found to increase hazard ratios for aortic valve stenosis (Kamstrup et al. 2014).

High levels of plasma Lp(a) can induce aortic valve stenosis through oxidation. Lp(a) consists of Apo(a), ApoB-100 and LDL-like particles. Lp(a) is known to play a primary role in carrying oxidized phospholipid (oxPL); in fact, ∼85% of circulating oxPL is carried by Lp(a) (Mahmut et al. 2014). oxPL was previously reported to activate endothelial cells, leading to monocyte trafficking and exacerbation of atherosclerosis (Leitinger 2005; Berliner et al. 2009). Moreover, inflammatory processes induced by oxPL recruit bone marrow-derived macrophages in a manner that depends on TLR2 activation. Because VICs express TLR2, oxPL may exacerbate aortic valve stenosis by inducing TLR2-mediated inflammatory processes and calcification (Meng et al. 2008; Kadl et al. 2011) In addition, Lp(a) can produce aortic valve stenosis by inducing autotaxin (ATX), which can further increase calcification. OxPL carried by Lp(a) can be converted to lysophosphatidylcholine (LPC) by the action of lipoprotein-associated phospholipase A2 (Lp-PLA2), which is known to be highly expressed in calcified valves (Mahmut et al. 2014). Once generated, LPC can be further processed to lysophosphatidic acid (LPA) by ATX. Bouchareb and colleagues were the first to demonstrate the relationship between Lp(a) and ATX and their roles in the progression of aortic valve stenosis. They showed that (1) Lp(a) can act as a transporter for ATX as well as oxPL, (2) ATX and Lp-PLA2 are co-localized in calcified valves, and (3) ATX is increased in patients with aortic valve stenosis.

ATX is mainly expressed in VICs and produces LPA, leading to activation of NF-κB together with LPC, produced by Lp-PLA2. Activation of NF-κB induces proinflammatory responses, including elevation of IL-6 and BMP2 leading to aortic valve stenosis (Bouchareb et al. 2015). This study suggests that Lp(a) can enhance aortic valve stenosis through various mechanisms.

Lp(a) also appears to be responsible for the poor efficacy of statins against aortic valve stenosis. Statins used to treat cardiovascular diseases, including atherosclerosis, have not been effective in improving aortic valve stenosis. Although statins, which inhibit HMG-CoA reductase, can decrease plasma LDL cholesterol, which is another risk factor for aortic valve stenosis, recent randomized placebo-controlled trials of statin treatment for CAVD have failed (Cowell et al. 2005; Chan et al. 2010). This may be explained by the fact that statins increase the levels of Lp(a) and oxPL/apoB (Fraley et al. 2009; Capoulade et al. 2015). Moreover, a meta-analysis has shown that statin treatment does not decrease LDL cholesterol in patients with the G allele of *LPA* rs10455872 (Donnelly et al. 2013). Taken together, these observations indicate that statin treatment can increase Lp(a), and the presence of a SNP in the *LPA* gene reduces the efficacy of statins, leading to ineffectiveness against aortic valve stenosis. A number of ongoing trials premised on the roles of Lp(a) in aortic valve stenosis seek to reduce the level of Lp(a) as a strategy for alleviating the disease. In this context, niacin and inhibitors of PCSK9 (proprotein convertase subtilisin/kexin type 9) are now being developed as anti-aortic valve stenosis therapeutic agents. In addition, a recently completed Phase I clinical trial of antisense therapy against Apo(a) showed dramatic reductions in Lp(a) (89%) and oxPL (93%) levels, raising hopes for the emergence of new therapeutic agents for aortic valve stenosis (Tsimikas et al. 2015; Graham et al. 2016; Yeang et al. 2016).

## Conclusions

**Figure 1.**
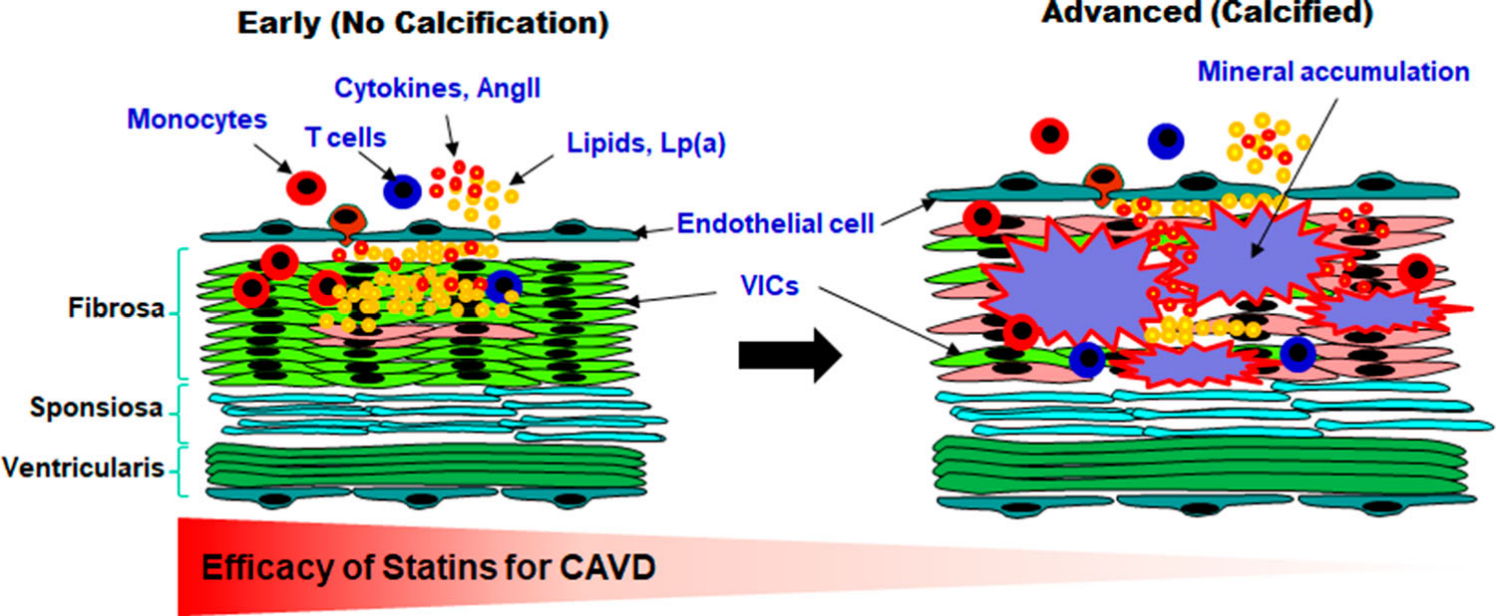
Various factors related in CAVD and Efficacy of statins against CAVD. Macrophages (differentiated from infiltrated monocytes) and infiltrated T cells produce proinflammatory cytokines which can induce valvular inflammation and calcification. VICs differentiate into myofibroblasts or osteoblast-like cells and aggravate valvular disease. Moreover myofibroblast-like VICs are also involved in AngII mediated valve thicking. In disease state, VECs upregulate cell adhesion molecules (CAMs) and these CAMs can recruit immune cells. Proinflammatory cytokines secreted from immune cells, VICs and VECs progress aortic valve into CAVD. Accumulating evidence suggests that statins may be effective against early stages of CAVD where calcification is absent or minimal, but not on advanced stages of the disease where calcification is more prominent. Lp(a) works as carrier of oxPL. Lp(a) and oxPL provoke TLR2-mediated or ATX-mediated inflammation and calcification. Also, statin treatment increases oxPL and its carrier Lp(a), which makes statin therapy is less effective against CAVD than against atherosclerosis. Thus, therapeutic approaches will necessarily differ depending on the stage of the disease.

In humans, CAVD and atherosclerosis progress slowly over a long period of time. In the early stages of both diseases, lesions are formed and progress through similar mechanism. In particular, early endothelial dysfunction and consequent inflammatory responses, including leukocyte recruitment, are involved in the initiation of both diseases. As the diseases progress, CAVD lesions develop more acellular regions characterized by lipid accumulation, calcification and mineralization. However, although advanced atherosclerotic lesions have large acellular regions showing lipid accumulation with a cholesterol cleft and mild mineralization, they also show greater accumulation of foam cells and inflammatory cells than CAVD lesions, a difference that is attributable to differences in the cellular composition between the aortic wall and valve. In particular, VICs, a major cell type in aortic valves, have a pivotal role in the progression of CAVD. These cells are activated in response to the initial inflammatory response and promote mineralization and bone formation – characteristic features of CAVD lesions. In advanced stages of CAVD, after mineralization and bone formation have progressed to a certain degree, lipid-lowering and anti-inflammatory statins are no longer effective (Figure 1). Therefore, patients at high risk for atherosclerosis or valvular disease may be prescribed statins in advance. Ultimately, understanding the molecular mechanisms responsible for mineralization in stenotic valves will be necessary to develop new therapeutic strategies for patients who have already undergone valve stenosis.
